# Effects of Salicylic Acid and Macro- and Micronutrients through Foliar and Soil Applications on the Agronomic Performance, Physiological Attributes, and Water Productivity of Wheat under Normal and Limited Irrigation in Dry Climatic Conditions

**DOI:** 10.3390/plants12122389

**Published:** 2023-06-20

**Authors:** Majed Alotaibi, Salah El-Hendawy, Nabil Mohammed, Bazel Alsamin, Nasser Al-Suhaibani, Yahya Refay

**Affiliations:** Department of Plant Production, College of Food and Agriculture Sciences, King Saud University, P.O. Box 2460, Riyadh 11451, Saudi Arabia

**Keywords:** arid countries, field conditions, grain yield, heatmap, limited irrigation, leaf pigments, relative water content, resource use efficiency, yield components

## Abstract

Ensuring food security with severe shortages of freshwater and drastic changes in climatic conditions in arid countries requires the urgent development of feasible and user-friendly strategies. Relatively little is known regarding the impacts of the co-application (Co-A) of salicylic acid (SA), macronutrients (Mac), and micronutrients (Mic) through foliar (F) and soil (S) application strategies on field crops under arid and semiarid climatic conditions. A two-year field experiment was designed to compare the impacts of seven (Co-A) treatments of this strategy, including a control, F_SA + Mic_, F_SA + Mac_, S_SA_ + F_Mic_, S_SA_ + F_SA + Mic_, S_SA + Mic_ + F_SA_, and S_SA + Mic_ + F_Mac + Mic_ on the agronomic performance, physiological attributes, and water productivity (WP) of wheat under normal (NI) and limited (LMI) irrigation conditions. The results reveal that the LMI treatment caused a significant reduction in various traits related to the growth (plant height, tiller and green leaf numbers, leaf area index, and shoot dry weight), physiology (relative water content and chlorophyll pigments), and yield components (spike length, grain weight and grain numbers per spike, thousand-grain weight, and harvest index) of wheat by 11.4–47.8%, 21.8–39.8%, and 16.4–42.3%, respectively, while WP increased by 13.3% compared to the NI treatment. The different Co-A treatments have shown a 0.2–23.7%, 3.6–26.7%, 2.3–21.6%, and 12.2–25.0% increase in various traits related to growth, physiology, yield, and WP, respectively, in comparison to the control treatment. The S_SA_+ F_SA + Mic_ was determined as the best treatment that achieved the best results for all studied traits under both irrigation conditions, followed by F_SA + Mic_ and S_SA + Mic_ + F_SA_ under LMI in addition to F_SA + Mac_ under NI conditions. It can be concluded that the Co-A of essential plant nutrients along with SA accomplished a feasible, profitable, and easy-to-use strategy to attenuate the negative impacts of deficit irrigation stress, along with the further improvement in the growth and production of wheat under NI conditions.

## 1. Introduction

Wheat (*Triticum aestivum* L.) is one of the mainstays for ensuring food security, especially in developing countries, where many people rely on wheat for their livelihoods. It is the staple food for approximately 25% of the world’s population and supplies a fifth of global food calories and protein [1]. It is cultivated under wide ranges of soil and climatic conditions and in many geographic regions [1,2]. Therefore, it is the most widely cultivated cereal crop in the world. In 2021, the wheat-cultivated area exceeded 220 million ha worldwide, which is 32% of the global cultivated cereal area. Additionally, the market size of wheat was valued at USD 127.7 billion and is forecasted to reach USD 169.1 billion by 2027 [2]. Wheat yields approximately 765 million metric tons of grain annually and more than 70% of this amount is used for food, while only 23% is used for livestock feed and in the industrial sector [2]. As the world population is increasing at an alarmingly fast rate, it is an urgent need to increase the current global wheat production by up to 50% by 2050 to meet future wheat requirements and ensure global food security [1]. Unfortunately, most regions of wheat production in several countries currently suffer from different types of drought stress due to abrupt climate changes that lead to an abrupt increase in temperature and severe periods of low precipitation. Water deficit stress is considered the most common factor causing a significant yield loss of the wheat crop in arid and semiarid countries, which may cause more than 50% yield losses compared to normal irrigation conditions [3,4].

There are several growth stages in the wheat crops that are very sensitive to water deficit stress, including tillering, flowering, and grain-filling stages. If the water deficit stress occurs at these stages, this can lead to disturbance in a number of physiological, morphological, and biochemical attributes in plants, which eventually hamper the development, growth, and production of crops through a substantial inhibition of cell division and enlargement rate, leaf and tiller development, leaf area, biomass accumulation, chlorophyll and relative water contents, photosynthesis efficiency, and different yield components [4,5,6,7,8]. Additionally, in general, the exposure of plants to water deficit stress leads to an imbalance in several plant nutrients, hormones, and growth regulators, an increase in leaf temperature, amount of reactive oxygen species, and the ratio of senescence, as well as a decrease in the translocation of metabolic components from source to sink [9,10,11]. Therefore, when applying irrigation water below the full requirements of the crop, it is obligatory to apply auxiliary approaches to lessen the negative impacts of this deficit irrigation on plant growth and production.

The alleviation of the negative impacts of water deficit stress in the field can be achieved by employing numerous approaches, including the usage of drought-tolerant genotypes, modern irrigation systems, and adopting site-specific agricultural practices, such as soil mulching, conservation tillage, modify planting patterns, and a proper scheduling of deficit irrigation [12,13,14]. However, these approaches are either economically challenging, labor-intensive, or need greater efforts over several years to obtain efficient results. When plants are under abiotic stresses, they develop complex and well-organized mechanisms to adapt themselves to such stresses and attenuate the negative impacts of these stresses on their growth and production. Biosynthesis and the accumulation of different osmolytes or compatible solutes, such as salicylic acid (SA), are considered one of these common mechanisms. SA is a promising compatible solute that can mitigate the negative impacts of water deficit stress through the regulation of various physiological and metabolic processes, including maintaining the stability and integrity of the cell membrane, restoring water and ion uptake, protecting cellular machinery from osmotic stress and oxidative damage, removing an excess level of reactive oxygen species (ROS), stimulating both enzymatic and non-enzymatic components of antioxidant defense system as well as regulating photosynthesis and transpiration rates, and stomatal conductance [4,15,16,17,18,19]. Therefore, previous studies have elucidated that the exogenous application of SA could be considered as a cheap, efficient, and easy-to-use approach for not only attenuating the negative impacts of deficit irrigation stress on the growth and production of crop plants but also enhancing the performance of plants under non-stress conditions [4,20,21,22]. 

Because the low nutritional status of soils is common in regions where crop plants are exposed to water deficit stress [9], previous studies also reported that the soil and foliar application of macronutrients (Mac) and micronutrients (Mic) is another promising approach to attenuate the negative impacts of deficit irrigation stress on the growth and production of crop plants. This is because several Mac and Mic are involved in a wide range of physiological process within the plant cells and several of these processes play a key role in enhancing the tolerance of plants to different abiotic stresses, including drought stress. Many studies reported that the application of Mac and Mic could successfully improve plant growth and alleviate the detrimental effects of water deficit stress on crop plants by maintaining photosynthetic activity, enzyme activity, and osmotic adjustment, preserving membrane integrity and stability, and chloroplast structure and functions, enhancing the plant’s defense against ROS, improving the plant’s ability to access soil moisture reserves, controlling water loss through the stomata, improving the development of the root system, improving the contents of proline and abscisic acids as well as they acting as a functional, structural, or regulatory cofactor of a large number of enzymes [9,23,24,25,26,27]. These dependent and independent roles of Mac and Mic reflect that the adequate management of plant nutrients may play important roles in enhancing the growth and production of crops under deficit irrigation stress. Karim and Rahman [9] investigated and reported that the soil application of Mac (NPK) and foliar application of Mic (Zn, B, and Mn) as well as the soil application of Mic in the early stage combined with foliar application in the late stage are promising approaches to the improve growth, production, and WP of wheat crops under drought stress conditions. Other studies also reported that the reduction in dry matter accumulation and grain yield of cereal crops, such as wheat, rice, and maize, due to drought stress under arid and semiarid conditions can be alleviated by the foliar application of Mic, such as zinc (Zn), boron (B), and manganese (Mn) [9,23,28,29,30]. 

In general, the leaves are the main organ in which the majority of physiological and biochemical processes occur. Therefore, previous comparative studies have reported that the application of SA and various plant nutrients on plants through the foliar spray (F) method is an effective and rapid approach to the relief of the negative effects of environmental stress on biochemical and physiological processes that occur in leaves as well as to improve the efficiency of nutrient use [29,31,32,33,34]. However, the F method, especially for plant nutrients, may cause leaf scorching, which ultimately destroys several physiological processes and therefore causes a considerable reduction in the growth and production of plants [33,34]. Furthermore, the efficiency of the exogenous application of SA and plant nutrients through the F method is greatly affected by leaf characteristics, air temperature, wind speed, light intensity, and amount of rain [29]. On the other hand, roots are the first plant organs to experience water deficit stress and perceive the signals of stress. The exposure of roots to water deficit stress leads to a significant decrease in their osmotic and water potentials, which in turn leads to a decrease in the turgor pressure of root cells and inhibition of root growth [35,36]. Therefore, the ability of roots to maintain and regulate their water relation and turgidity through an osmotic adjustment (OA) mechanism leads to some degree of drought tolerance and improves water uptake. The accumulation of osmolytes or compatible solutes, such as SA, and different plant nutrients in the root cells plays a vital role not only in the regulation of OA of roots but also protects the root system of plants from ROS by up-regulating the activity of enzymes associated with the antioxidant defense system under water deficit stress, which ultimately improves root growth and water uptake [27,36]. Therefore, the application of SA and various plant nutrients to plants through the soil (S) method can be considered an effective approach to significantly increase their concentration in root cells. Based on the aforementioned points, we hypothesized that the application of SA, Mac, and Mic to plants through only one method of application may not be effective for alleviating the negative impacts of water deficit stress and improving the growth and production of wheat crops under conditions of deficit irrigation. In addition, the application of SA by the F method has been studied in much more depth than that by the S method, although studies using the S method for applying SA are also necessary. Furthermore, the relative efficacy of the F and S application methods of SA combined with plant nutrients has not been well tested in the field under dry climatic conditions. Thus, the main objective of this study is to evaluate the effectiveness of the F and S application methods of SA combined with Mac and Mic on the agronomic performance, physiological attributes, and WP of wheat crops under full and deficit irrigation conditions in an arid agro-ecosystem field.

## 2. Results

### 2.1. Effects of Experimental Factors on Growth Parameters

Only the leaf area index (LAI) was significantly influenced by the main impacts of season (S), whereas irrigation factor (IR) and the co-application of SA, Mac, and Mic factor (Co-A) had a considerable impact on all growth parameters (Table 1). The interaction of season in IR and season in Co-A was insignificant for all the studied growth parameters, except green leaf number (GLN) and LAI, whereas the interaction of IR in Co-A was significant for all the growth parameters at *p* ≤ 0.05 or 0.01, except plant height (PH) and tiller number (TN). Only GLN was affected by the triple interaction of season, IR, and Co-A (Table 1). 

Wheat plants subjected to limited irrigation (LMI) exhibited a significant reduction in all growth parameters compared to the normal irrigation (NI) treatment. The PH, TN, GLN, LAI, shoot fresh weight (SFW), and shoot dry weight (SDW) under LMI were 11.4%, 28.7%, 42.5%, 47.8%, 31.5%, and 29.2% lower than those under the NI treatment, respectively (Table 1). Contrarily, in the wheat plants treated with different Co-A treatments, all the aforementioned growth parameters were significantly enhanced when compared to the untreated plants (control treatment). Generally, the S application of SA combined with the F application of SA and Mic (S_SA_ + F_SA + Mic_) exhibited the highest values for all growth parameters, followed by the F application of SA and Mic alone (F_SA + Mic_) or the S application of SA and Mic combined with the F application of SA (S_SA + Mic_ + F_SA_). The application of SA and Mic through the F method (F_SA + Mac_) exhibited higher GLN, LAI, SFW, and SDW than those of the application of SA through the S method and Mic through the F method (S_SA_ + F_Mic_) or the application of SA and Mic through the S method and Mac and Mic through the F method (S_SA + Mic_ + F_Mac + Mic_). The untreated plants (control treatment) always exhibited the lowest values of all growth parameters, and the different Co-A treatments caused significant increases in PH by 0.2–4.3%, TN by 7.5–13.9%, GLN by 10.3–23.7, LAI by 8.1–22.1, SFW by 4.9–16.3%, and SDW by 8.0–19.6%, when compared with the control treatment (Table 1).

**Figure 1:**
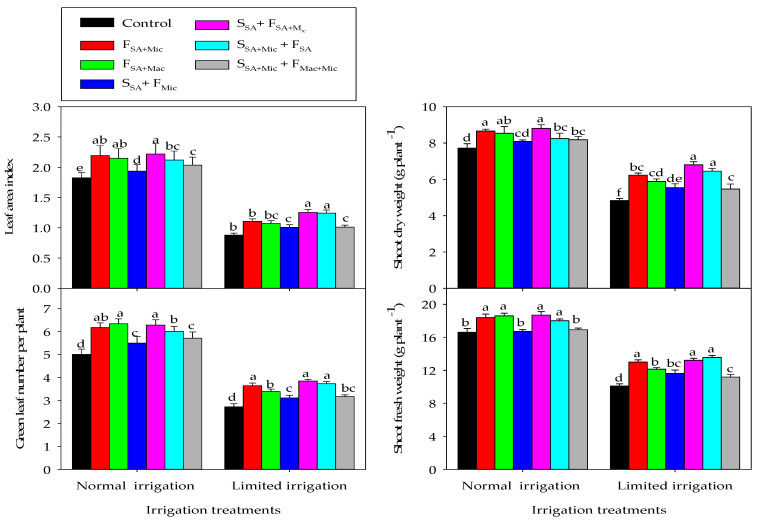
Interactive effect of irrigation and the co-application of salicylic acid (SA), macronutrients (Mac), and micronutrients (Mic) through foliar (F) and soil (S) application methods on different growth parameters over two growing seasons. Bars sharing the same letter are not significantly different at the 0.05 level according to Tukey’s test. The bars show the standard deviation (n = 3).

The response of all studied growth parameters to the different Co-A treatments varied with the IR treatments. In the NI treatment, the different growth parameters in different Co-A treatments in most cases followed the order of S_SA_ + F_SA + Mic_ ≈ F_SA + Mac_ ≈ F_SA + Mic_ > S_SA + Mic_ + F_SA_ > S_SA + Mic_ + F_Mac + Mic_ ≈ S_SA_ + F_Mic_ > control, whereas they ranked in the order of S_SA_ + F_SA + Mic_ ≈ S_SA + Mic_ + F_SA_ > F_SA + Mic_ > F_SA + Mac_ > S_SA_ + F_Mic_ ≈ S_SA + Mic_ + F_Mac + Mic_ > control under the LMI treatment (Figure 1). These results indicate that the S_SA_ + F_SA + Mic_ was the best treatment and exhibited the highest values for most growth parameters under both IR treatments, followed by F_SA + Mac_ and F_SA + Mic_ under the NI treatment and S_SA + Mic_ + F_SA_ under the LMI treatment. The best three Co-A treatments under NI conditions caused significant increases in GLN by 18.9–21.0, LAI by 15.0–17.7, SFW by 3.7–5.5%, and SDW by 9.6–12.3%, whereas the best two Co-A treatments under LMI conditions caused significant increases in GLN by 26.9–29.2, LAI by 29.2–29.9, SFW by 10.6–12.9%, and SDW by 25.1–29.0% compared with the control treatment (Figure 1). 

### 2.2. Effects of Experimental Factors on Physiological Parameters

Table 2 shows the response of different physiological parameters to the main effects of S, IR, Co-A, and their different interactions. The S, IR, and Co-A had a considerable effect on all physiological parameters. All physiological parameters were also significantly affected by the interaction of S with IR, S with Co-A except relative water content (RWC), and IR with Co-A. Only the chlorophyll-b content (Chl-b) was affected by the triple interaction of S, IR, and Co-A (Table 2). 

The LMI treatment significantly reduced the RWC by 21.8%, chlorophyll-a content (Chl-a) by 38.6%, Chl-b by 39.8%, and total chlorophyll content (Chlt) by 39.2%, in comparison to the NI treatment (Table 2). However, the wheat plants treated with different Co-A treatments showed a 3.6–10.5%, 15.0–26.7%, 11.5–26.7%, and 13.5–26.2% increase in RWC, Chl-a, Chl-b, and Chlt, respectively, in comparison to the control treatment (Table 2). Generally, S_SA_ + F_SA + Mic_ was the best treatment for achieving the highest values for all physiological parameters; surpassing the control treatment by 10.5%, 26.7%, 26.7%, and 26.2% for RWC, Chl-a, Chl-b, and Chlt, respectively. The F_SA + Mic_, F_SA + Mac_, and S_SA + Mic_ + F_SA_ produced comparable values for different physiological parameters as did the best treatment (S_SA_ + F_SA + Mic_), and these treatments showed a 7.4–9.9%, 23.9–24.4%, 21.0–24.4%, and 23.5–23.8% increase in RWC, Chl-a, Chl-b, and Chlt, respectively, in comparison to the control treatment. Although the S_SA_ + F_Mic_ and S_SA + Mic_ + F_Mac + Mic_ exhibited the lowest values of all physiological parameters among the different Co-A treatments, the values of RWC, Chl-a, Chl-b, and Chlt in these treatments were 3.6–4.8%, 15.0–17.1%, 11.5–12.8%, and 13.5–15.0%, respectively, than those in the control treatment (Table 2).

**Figure 2:**
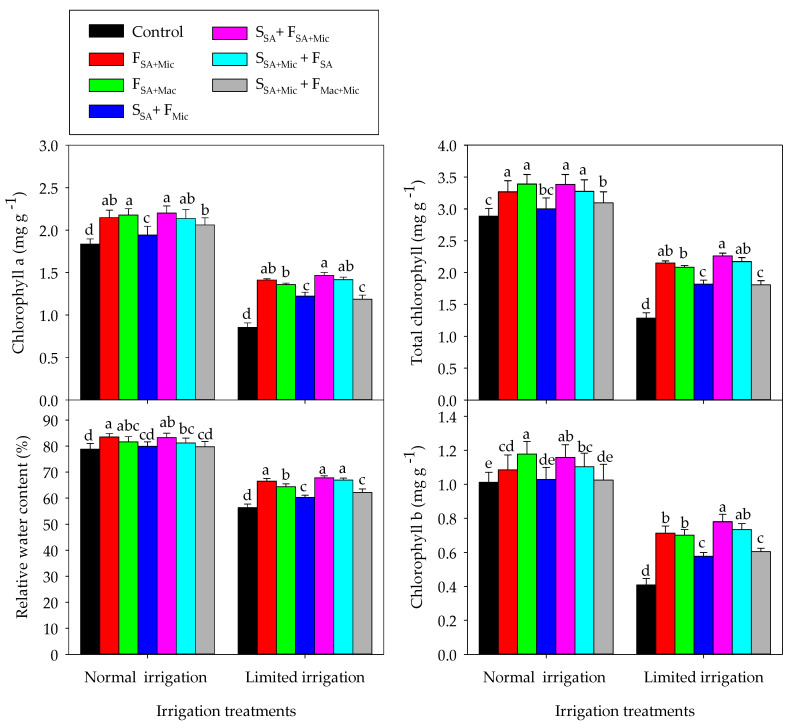
Interactive effect of irrigation and the co-application of salicylic acid (SA), macronutrients (Mac), and micronutrients (Mic) through foliar (F) and soil (S) application methods on different physiological parameters over two growing seasons. Bars sharing the same letter are not significantly different at the 0.05 level according to Tukey’s test. The bars show the standard deviation (*n* = 3).

The response of different physiological parameters to the different Co-A treatments varied also with the IR treatment, as shown in Figure 2. In general, the different Co-A treatments improved all physiological parameters in comparison to the control treatment under either NI or LMI conditions; however, these Co-A treatments were more effective in enhancing the different physiological parameters under LMI than NI conditions. Compared with the control treatment under each IR treatment, the values of RWC, Chl-a, Chl-b, and Chlt increased by 1.1–5.6%, 5.5–16.6%, 1.2–14.1%, and 3.8–14.9%, respectively, under NI conditions and by 6.6–16.9%, 27.9–41.8%, 29.2–47.6%, and 29.0–43.3%, respectively, under LMI conditions (Figure 2). Finally, F_SA + Mic_, S_SA_+ F_SA + Mic_, and S_SA + Mic_ + F_SA_ were the best treatments for achieving the highest values for all physiological parameters under both IR conditions, whereas S_SA_+ F_Mic_ and S_SA + Mic_ + F_Mac + Mic_ were less effective than the other Co-A treatments for enhancing the physiological parameters also under both IR conditions (Figure 2).

### 2.3. Effects of Experimental Factors on Yield Parameters and Water Productivity

All yield parameters and WP were significantly affected by the main impacts of IR and Co-A treatments as well as the interaction between both, with the exception of spike length (SL), which was not significantly affected by the main impacts of Co-A and the interaction between IR and Co-A (Table 3). Contrarily, all yield parameters and WP were not significantly affected by the main impacts of S, the interaction between S and IR except thousand-grain weight (TGW), and the interaction between S and Co-A. Only the TGW was significantly affected by the triple interaction of S, IR, and Co-A (Table 3). 

The LMI treatment caused a significant reduction in all yield parameters, while it improved WP. Compared with the NI treatment, the values of SL, grain weight per spike (GWS), grain number per spike (GNS), TGW, grain yield (GY), biological yield (BY), and harvest index (HI) decreased by 16.4%, 38.7%, 23.8%, 19.7%, 42.3%, 30.6%, and 17.2%, respectively. Meanwhile, WP values were higher by 13.3% under LMI than NI treatment (Table 3). Contrarily, the plants treated with the different Co-A treatments seemed to be superior in enhancing yield parameters and WP compared with the untreated plants. The different Co-A treatments showed a 8.0–20.3%, 2.3–10.8%, 5.2–12.6%, 9.9–21.6%, 5.4–15.0%, 5.8–9.4%, and 12.2–25.0% increase in GWS, GNS, TGW, GY, BY, HI, and WP, respectively, in comparison to the control treatment (Table 3). S_SA_ + F_SA + Mic_ was the best treatment for achieving the highest values for all yield parameters and WP, followed by the S_SA + Mic_ + F_SA_ treatment. S_SA_+ F_Mic_ and S_SA + Mic_ + F_Mac + Mic_ were less effective than the other Co-A treatments for enhancing the yield parameters and WP, but they still enhanced the GWS, GNS, TGW, GY, BY, HI, and WP by 8.0–8.1%, 2.3–4.3%, 5.2–7.2%, 9.9–10.2%, 5.4–5.7%, 5.8–6.3%, and 12.2–12.9%, respectively, in comparison to the control treatment (Table 3).

**Figure 3:**
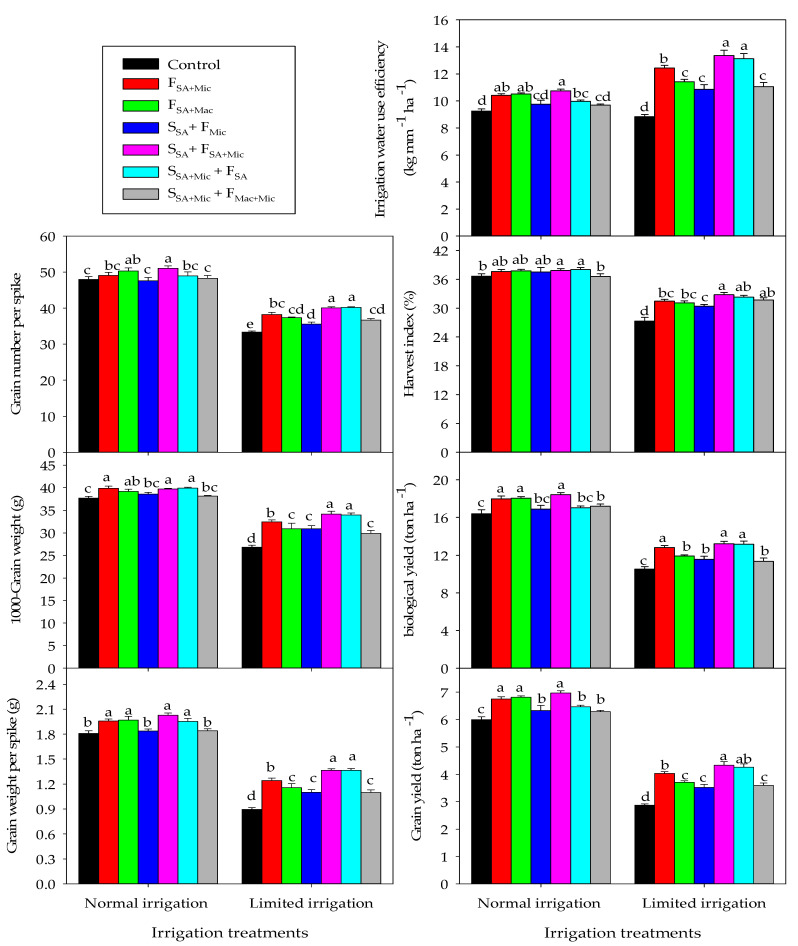
Interactive effect of irrigation and the co-application of salicylic acid (SA), macronutrients (Mac), and micronutrients (Mic) through foliar (F) and soil (S) application methods on different yield, yield components, and water productivity over two growing seasons. Bars sharing the same letter are not significantly different at the 0.05 level according to Tukey’s test. The bars show the standard deviation (n = 3).

Under NI conditions, the highest values for yield parameters and WP were achieved with F_SA + Mic_, F_SA + Mac_, and S_SA_+ F_SA + Mic_. These three treatments enhanced GWS by 7.7–10.9%, GNS by 2.3–6.1%, TGW by 3.8–5.5%, GY by 11.2–13.9%, BY by 8.8–11.1%, HI by 2.5–3.1%, and WP by 11.2–13.9%as compared to the control treatment. S_SA + Mic_ + F_SA_ produced comparable values for GWS, TGW, and HI as did the above mentioned three treatments (Figure 3). Under LMI conditions, the highest values for yield parameters and WP were obtained with the S_SA_+ F_SA + Mic_ and S_SA + Mic_ + F_SA_ treatments, followed by the F_SA + Mic_ and F_SA + Mac_ treatments. The former two treatments enhanced SL, GWS, GNS, TGW, GY, BY, HI, and WP by 2.2–3.7%, 34.2–34.4%, 16.7–17.1%, 20.9–21.4%, 32.6–33.8%, 20.1–20.4%, 15.4–16.7%, and 32.6–33.8%, whereas the latter two treatments enhanced all the parameters mentioned above by 2.4–4.1%, 22.6–22.9%, 10.8–12.8%, 13.3–17.4%, 22.6–28.9%, 11.7–17.9%, 12.2–13.2%, and 22.6–28.9%, respectively, as compared to the control treatment (Figure 3). 

### 2.4. Pearson’s Correlation Coefficient between all Studied Parameters under Normal and Limited Irrigation Conditions

Pearson’s correlations analysis was performed based on the main effects of different Co-A treatments over two growing seasons to show the relationship between the different parameters of growth, physiological, yield components, grain yield, and WP of wheat under each IR treatment (Table 4). Generally, the correlations among the studied parameters under the LMI treatment were higher than those under the NI treatment. PH, TN, SL, and HI had non-significant correlations with all the studied parameters under the NI treatment, while they exhibited a strong and positive correlation with all parameters under the LMI treatment. All growth and physiological parameters exhibited strong and positive correlations with GY and WP (r ≈ 0.81–1.00) under both IR treatments except PH and TN under the NI treatment (Table 4). The RWC and chlorophyll pigments exhibited strong and positive correlations with almost all growth and yield parameters under both IR treatments (Table 4). 

### 2.5. Heatmap Analysis for Providing an Overall Picture of the Response of Different Parameters of Wheat to Various Co-A Treatments under Each Irrigation Treatment 

**Figure 4:**
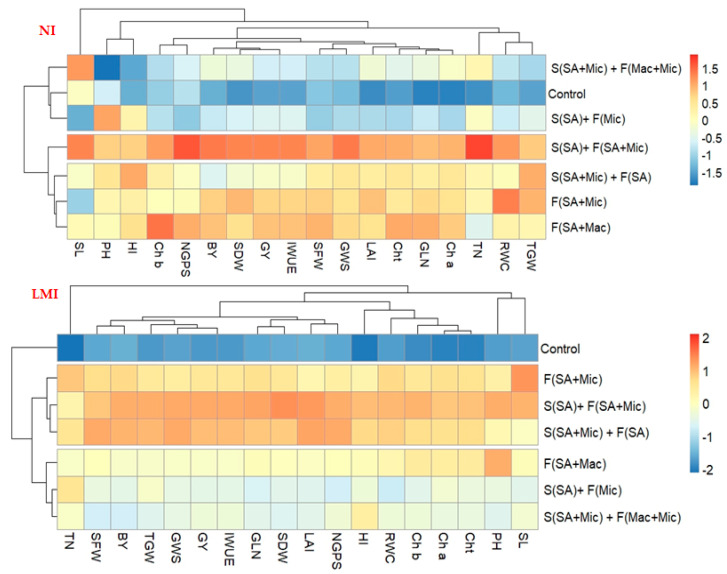
Heatmap cluster analysis describing the response of the different studied parameters of wheat to the various co-applications (Co-A) of salicylic acid (SA), macronutrients (Mac), and micronutrients (Mic) through foliar (F) and soil (S) application methods under normal (NI) and limited (LMI) irrigation treatments. Abbreviations of PH, TN, GLN, LAI, SFW, SDW, RWC, Chl-a, Chl-b, Chlt, SL, GWS, GNS, TGW, GY, BY, HI, and WP are plant height, tiller number per plant, green leaf number per plant, leaf area index, shoot fresh weight, shoot dry weight, relative water content, chlorophyll-a, chlorophyll-b, total chlorophyll content, spike length, grain weight per spike, grain number per spike, thousand-grain weight, grain yield per ha, biological yield per ha, harvest index, and water productivity, respectively.

The heatmap clustering analysis was conducted on the data of all parameters (18 parameters) and seven Co-A treatments under each irrigation treatment (Figure 4). As seen from the figure, the Co-A treatments were grouped in three clusters under each irrigation treatment, whereas the eighteen parameters were grouped in four and five clusters under NI and LMI conditions, respectively. Under the NI treatment, the S_SA_+ F_SA + Mic_ treatment was clustered alone in one group and exhibited the highest values for all studied parameters. F_SA + Mic_, F_SA + Mac_, and S_SA + Mic_ + F_SA_ were grouped together in one cluster and also displayed the highest values for almost all the studied parameters. Furthermore, S_SA_+ F_Mic_ and S_SA + Mic_ + F_Mac + Mic_ were grouped together with the control treatment in one cluster and exhibited medium and the lowest values for almost all the studied parameters (Figure 4). Under the LMI treatment, FS_A + Mic_, S_SA_+ F_SA + Mic_, and S_SA + Mic_ + F_SA_ were grouped together in one cluster and exhibited the highest values for the different parameters, while the control treatment was clustered alone in one group and exhibited the lowest values for all the studied parameters. F_SA + Mac_, S_SA_+ F_Mic_, and S_SA + Mic_ + F_Mac + Mic_ were grouped together in one cluster and exhibited medium values for all the studied parameters (Figure 4). 

### 2.6. Economic Budgeting 

Under the NI treatment, F_SA + Mic_ and F_SA + Mac_ resulted in the highest monetary returns, followed by S_SA_+ F_SA + Mic_, while S_SA_+ F_Mic_, S_SA + Mic_ + F_SA_, and S_SA + Mic_ + F_Mac + Mic_ recorded the highest costs as compared to the control treatment (Table 5). Under the LMI treatment, all Co-A treatments, except S_SA + Mic_ + F_Mac + Mic_, recorded more monetary returns than the control treatment. Additionally, S_SA_+ F_Mic_, S_SA + Mic_ + F_SA_, and S_SA + Mic_ + F_Mac + Mic_ under the NI treatment as well as S_SA + Mic_ + F_Mac + Mic_ under the LMI treatment recorded more total costs than the control treatment (Table 5).

## 3. Discussion

To ensure the future food security of a rapidly increasing population, it is necessary not only to minimize the negative effects of abiotic stresses, such as water deficit, on field crop production, but also to strive to maximize their production under normal conditions. In general, a balanced supply of essential plant nutrients to the wheat crop is vital to enhance their growth and production not only under water deficit stress conditions but also under normal irrigation, particularly when grown in arid and semiarid climates where multiple nutrient deficiencies become the growth- and production-limiting factor under these climates due to high pH, low organic matter, and excessive bicarbonate [4,37]. In this experiment, all treatments that involved the foliar application of plant nutrients significantly improved the growth (PH, TN, GLN, LAI, SFW, and SDW), physiological (RWC, Chl-a, Chl-b, and Chlt), and yield parameters (SL, GWS, GNS, TGW, GY, BY, and HI) as well as WP of wheat in comparison to the control treatment under both NI and LMI conditions (Table 1, Table 2 and Table 3 and Figure 1, Figure 2 and Figure 3). These results reveal that the foliar application of plant nutrients is necessary to enhance wheat performance under normal irrigation in dryland conditions, and is also particularly important in alleviating the negative impacts of limited water irrigation on the growth, physiology, and yield parameters of wheat. The increase in all parameters of wheat with the foliar application of essential nutrients under NI conditions can be attributed to the fact that this practice helps to supply nutrients to plants more quickly than the soil application, avoids the depletion of essential nutrients in leaves, permits the correction of nutrient deficiencies in a short time, and accelerates the absorption of nutrients. Most importantly, the nutrient uptake by this practice is not affected by root health and soil physicochemical properties and therefore increases the efficiency of plant nutrient use [27,29,38]. All of the above advantages of the foliar spray method lead to delayed leaf senescence, increased LAI, enhanced chlorophyll biosynthesis, improved solar radiation utilization, prolonged photosynthetic rate, and increased transport rate of photosynthetic products from leaves to the developing grains [27,37,38,39,40,41]. These abovementioned positive results of the application of plant nutrients through the F method may elucidate why treatments involving the foliar application of Mac and Mic nutrients under NI conditions produced higher growth, physiological attributes, yield, and yield components than the control treatment (untreated plants), as shown in Figure 1, Figure 2 and Figure 3, as well as it may explain also why the different growth parameters, such as GLN, LAI, SFW, and SDW, and photosynthetic pigments, such as Chl-a, Chl-b, and Chlt, exhibited strong and positive correlations with yield and yield components, as shown in Table 4. Previous studies have also reported significant impacts of the F method of Mac and Mic nutrients on different growth parameters and concentrations of chlorophyll and carotenoids under normal irrigation conditions in arid and semiarid climates, which ultimately enhanced yield and yield components [34,37,39,42,43]. For example, Dass et al. (2022) found that the foliar application of Mac (urea_2%_ and NPK_2%_) and Mic (Zn0.5% and B0.5%) improved the GY of soybean by 16.6–37.8% in comparison to no-foliar nutrition under a semiarid climate. Amanullah et al. (2021) also reported that, under normal irrigation, the foliar application of Mic (Zn + B) and Mac (P + K) nutrients alone and in various combinations produced the highest values for SL, GNS, TGW, number of spikelets per spike, and GY of wheat in calcareous soils under arid and semiarid climates. 

In this study, the LMI treatment significantly reduced all the studied parameters compared with the NI treatment (Figure 1, Figure 2 and Figure 3). This is because water deficit stress induces substantial alterations in the morphological, physiological, and biochemical characteristics of plant leaves, including lowering photosynthetic rate, stomatal conductance, expansion and division of cells, enzyme activity, chlorophyll biosynthesis, RWC, and biomass accumulation. It also induces oxidative damage due to the excess generation of ROS, which results in the altered function of key metabolic pathways, such as photosynthesis, chlorophyll biosynthesis, and mineral uptake and assimilation. It also restricts root growth, which ultimately restricts essential nutrient uptake even with they are available in the soil [10,11,44,45,46]. The aforementioned disturbances in different morphological, physiological, and biochemical characteristics of the plants may explain why the LMI treatment significantly reduced the different studied parameters of wheat compared to the NI treatment, as shown in Figure 1, Figure 2 and Figure 3. However, the treatments involving the foliar application of plant nutrients exhibited higher growth, physiological attributes, yield, yield components, and WP than the control treatment (no foliar application), as shown in Figure 1, Figure 2 and Figure 3. In addition, strong and positive correlations were observed between all the studied parameters under the LMI treatment (Table 4). These results also indicate that the supply of plant nutrients via the F method is of paramount importance to mitigate the negative impact of water deficit stress. Thus, this practice can be considered as an effective and simple approach to attenuate the negative impact of deficit water stress on the growth and production of wheat in arid and semiarid climates. The reason for this can be attributed to the fact that the uptake of nutrients through this practice is completely independent of the water content of the soil, the physical and chemical properties of the soil, and the activities of the roots. Additionally, this practice helps to introduce the essential plant nutrients required for the plant directly into the leaves, thus helping to speed up their absorption rates and concentrations in the cells sufficiently. This helps various plant nutrients to rapidly relieve the negative impact of water deficit stress on the various morphological, physiological, and biochemical processes inside the plant system. For example, the presence of nitrogen in plant cells under water deficit stress helps in lowering ROS concentrations by triggering proline accumulation and enzymatic antioxidant activities, reducing leaf senescence, enhancing leaf chlorophyll contents, accelerating cell synthesis and expansion of plant cells and xylem tissues, facilitating osmoregulation, carbon partitioning, cellular membrane stability, and carbohydrate build-up, and maintaining optimum leaf RWC by enhancing the plasticity and ability of roots to extract water from the soil [27,47,48]. Phosphorus under water deficit stress helps in enhancing root architecture and proliferation in the soil by stimulating root volume and hydraulic conductance and maintaining the cell turgidity and cell membrane stability through the acceleration of net photosynthesis and stomatal conductance. It also plays many vital roles in energy transfer, photosynthesis processes, enzyme activation, metabolism and movement of carbohydrates and lipids, membrane structure, chlorophyll biosynthesis, and osmolytes accumulation [27,49,50]. Likewise, potassium under water stress plays a vital role in various physiological and metabolic functions of a plant system, such as stomatal opening and closing, osmotic adjustment and turgor pressure regulation, cytoplasmic homeostasis, photosynthates translocation, protein and carbohydrate synthesis, and the activations of a wide range of enzymes that regulate water use efficiency [51,52,53]. Micronutrients, such as Zn and Mn, have also proved their role in alleviating water deficit stress. An adequate Zn supply under water deficit stress improves the activity of the antioxidant system, regulates membrane permeability, facilitates ion transport, activations of antioxidant substances, enhances photosynthetic efficiency and water use efficiency, and improves photosynthetic pigments, stomatal conductance, leaf RWC, and osmolytes accumulation [26,27]. Therefore, a sufficient essential plant nutrient supply under field conditions in arid and semiarid climates, which can be achieved through the F method, helps in alleviating the negative impact of limited water irrigation on the growth and production of wheat crops through the modulation of multiple morphological, physiological, and biochemical processes inside the plant system. Moreover, the results of this study confirm that the F practice for plant nutrients could be considered a low-cost and sustainable way for enhancing the growth and production of wheat under LMI conditions. The results of this study are in line with those of Mahmoodi et al. [39] in rice, Akhtar et al. [52] in durum wheat, and Deswal and Pandurangam [42] in maize, who reported that the negative effects of water deficit stress on various growth, physiological, and yield parameters were profoundly attenuated by the foliar application of various plant nutrients. 

The results of this study also found that the S_SA_+ F_SA + Mic_ treatment has been noticed to be very effective in enhancing the growth, physiological, and yield parameters of wheat followed by the S_SA + Mic_ + F_SA_, F_SA + Mic_, and F_SA + Mac_ treatments (Table 1, Table 2 and Table 3). These treatments are also effective for enhancing the performance of wheat under NI conditions and curbing the negative consequences of water-deficit-induced stress on wheat performance under LMI conditions (Figure 1, Figure 2, Figure 3 and Figure 4). These results indicate that the integration of SA and plant nutrients also plays a positive role in enhancing wheat performance under both normal and water deficit stress conditions. In addition, the application of SA through the S and F methods also plays a vital role in improving the growth and production of wheat under both normal and water deficit stress conditions. These results reflect that the combination of the S and F application methods for SA and plant nutrients had a synergistic or a stimulatory effect on wheat performance under NI conditions as well as having a protective role in curbing the negative effects of water deficit stress on the growth and production of wheat under LMI conditions. Since the leaves are the primary plant organ in which the majority of physiological and biochemical processes happen, and the roots are the first plant organ exposed to water deficit stress, this may explain why the practice of the combination of the S and F application methods for SA and plant nutrients is effective for curbing the negative impacts of water deficit stress and improving wheat performance under normal conditions. The role of plant nutrients in enhancing growth, physiological attributes, yield, and yield components under both normal and water deficit stress conditions was explained above. SA is also a promising phytohormone that plays a vital role in improving the growth and production of wheat crops under both normal and water deficit stress conditions. This finding could be due to SA helping to regulate the stomata pore opening, thus improving the photosynthesis rate and stomatal conductance, which ultimately leads to enhancing the growth and production of plants under both normal and stress conditions [16,17,21,22,54]. It activates the biosynthesis of several important enzymes that are involved in a wide range of different physiological and biochemical processes, thus improving the growth and production of plants under both normal and stress conditions [55]. It also plays a key role in enhancing the division and elongation of cells, the contents of leaf chlorophyll, as well as delaying leaf senescence, thus enhancing and prolonging the photosynthesis rate, which ultimately leads to enhancing the growth and production of plants under both normal and stress conditions [54,56]. SA is also involved in diminishing the unnecessary loss of water in plants by reducing the transpiration rate through regulating stomatal closure, thus ultimately leading to an improved plant water status [57]. It also plays a vital role in enhancing root growth, i.e., root elongation, root branching, and adventitious rooting, thus enabling the roots to extract water and essential plant nutrients from deeper soil layers, which eventually leads to improving plant biomass and increasing yield traits under both normal and stress conditions [58]. These positive effects of SA might explain why the exogenous application of SA through the S and F methods seems to be a beneficial approach in coping with water deficit stress as well as in improving the growth and production of wheat crops under normal and stress conditions. Similarly, the improved growth, physiological attributes, yield, and yield components of different field crops grown under normal and stress conditions after the foliar application of SA have been reported by several previous studies [4,18,21,59]. However, the effects of SA application through the soil on the growth, physiological attributes, yield, and yield components of wheat under normal and stress conditions have not been studied to date.

## 4. Materials and Methods

### 4.1. Experimental Design, Site, Conditions, and Treatments 

**Figure 5:**
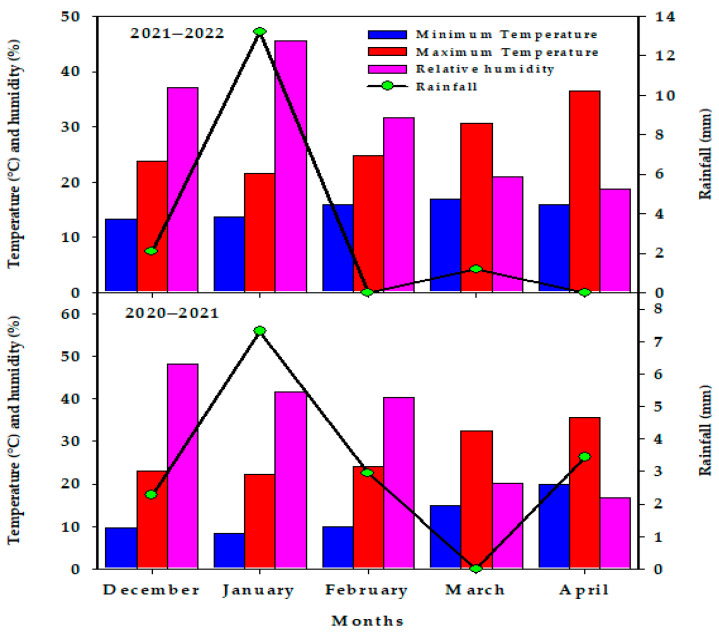
Monthly averages of the minimum/maximum temperature, relative humidity, and rainfall during the two growing seasons of wheat at the Research Station in the Riyadh region, Saudi Arabia.

In two cropping seasons (2020–2021 and 2021–2022) from December to April, a split-plot experiment was conducted based on a randomized complete block design with three replications. The field experiment was conducted at the Dierab Research Station of the College of Food and Agriculture Sciences, King Saud University in Riyadh, Saudi Arabia, at 46°39′ E and 24°25′ N, and 570 m above sea level. Figure 5 shows the monthly averages of minimum/maximum temperature, relative humidity, and rainfall during the two cropping seasons of wheat at the Research Station, while Table 6 shows the different physicochemical properties of the soil collected from the study site. The two irrigation treatments (IR) were distributed in the main plots, while the seven Co-A treatments were randomly distributed in the subplots. The first irrigation treatment represents NI (irrigation with 100% of the estimated crop evapotranspiration, ETc), while the second one represents the LMI treatment (irrigation with 50% ETc). The quantity of irrigation water required for the NI treatment was calculated based on the reference evapotranspiration rate (ETo) and crop coefficient (Kc). The ETo was calculated according to the modified Penman–Monteith equation using the daily climatic data of the experimental site, while the Kc values of spring wheat reported in the FAO-56 were used after adjustment based on the relative humidity and wind speed at the experimental site [60]. Based on this calculation, approximately 6470 and 6500 m^3^ ha^−1^ were applied for the NI treatment in the first and second seasons, respectively. Half of this amount was applied for the LMI treatment. A low-pressure-modified surface irrigation system was used to apply the irrigation water, as outlined by [14]. 

**Figure 6:**
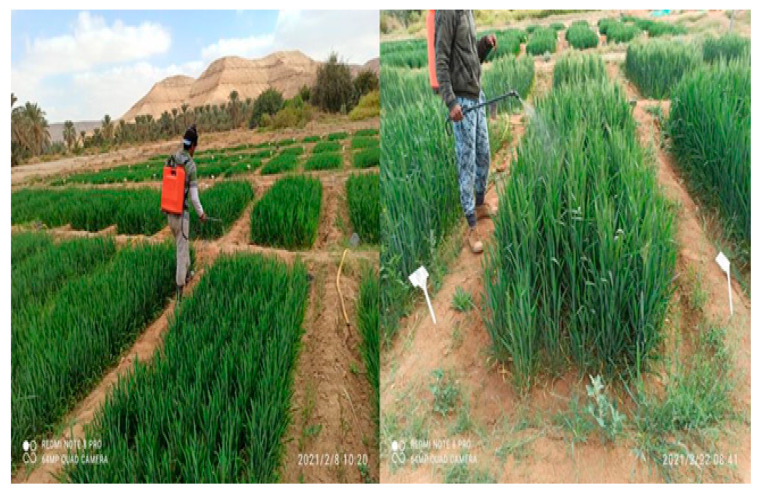
Plot layouts of the application of salicylic acid and plant nutrients through the foliar spray method.

The seven Co-A treatments included the co-application of SA, Mac, and Mic through the F and S application methods, i.e., F_SA + Mic_, F_SA + Mac_, S_SA_ + F_Mic_, S_SA_ + F_SA + Mic_, S_SA + Mic_ + F_SA_, and S_SA + Mic_ + F_Mac + Mic_. The S application of SA and Mic (Zn and Mn) was conducted once at the phenological Zadoks scale 29 (tillering stage), while the F application of SA, Mac (N, P, and K), and Mic was conducted twice at the phenological Zadoks scale 33 (stem elongation) and 51 (ear emergence) [61]. The S application of SA, Zn, and Mn was applied at a rate of 3, 20, and 15 kg ha^−1^, respectively. SA and Mn were exogenously sprayed at the concentrations of 2.0 mM and 0.5%, respectively, while Zn, N, P, and K were exogenously sprayed at the concentration of 1.0% for each. 2-Hydroxybenzoic acid [C_6_H_4_(OH) COOH], zinc sulfate (ZnSO_4_), manganese sulfate (MnSO_4_), aqueous solutions of urea [CO(NH_2_)_2_], dipotassium phosphate (KH_2_PO_4_), and potassium sulfate (K_2_SO_4_) were used as sources for SA, Zn, Mn, N, P, and K, respectively. To guarantee an effective penetration of SA and plant nutrients in wheat leaves, a small amount of Tween-20 (C_58_ H_114_ O_26_; 0.1% v/v) as a nonionic polyoxymethylene agent was mixed with the spraying solutions. Thereafter, the solutions of SA and plant nutrients were sprayed directly onto the leaves until the whole leaf surface of the plants was wet using a backpack pressurized sprayer (16 L) (Figure 6). 

### 4.2. Agronomical Management Practices

After plowing and leveling the soil, the experimental field was divided into two main plots (60 m × 4 m each). After that, each main plot was divided into twenty-one subplots (1.4 m × 4 m each), with a 60 cm buffer zone between two adjacent subplots. During seedbed preparation, calcium superphosphate (15.5% P_2_O_5_) was incorporated into the soil at a rate of 31 kg P_2_O_5_ ha^−1^ and then the seeds of wheat (*Triticum aestivum* L.) cultivar Summit were sown manually in each subplot in seven rows with 0.2 m spacing at a seeding rate of 15 g m^2^. The seeds were sown on 8 December 2020 and 1 December 2021. Potassium (K) in the form of potassium sulfate (48% K_2_O) and nitrogen (N) in the form of urea (46% N) were applied at a rate of 60 kg K_2_O ha^−1^ and 180 kg N ha^−1^ in two and three equal doses, respectively. The first two doses of both fertilizers were applied at the four-leaf stage (Zadoks scale 14) and tillering stage (Zadoks scale 29), while the last dose of N was applied at the booting stage (Zadoks scale 45). Other agronomic practices, such as weeding and disease control, were conducted consistently based on the local recommendations. 

### 4.3. Data Recording and Related Procedures

#### 4.3.1. Growth Parameters

At the mid-flowering stage (a Zadoks scale 65: approximately 100 days after sowing), ten plants from the second and sixth rows of each subplot were randomly selected to determine the different growth indicators. All plants were immediately weighed using a digital balance to determine the SFW. The PH was measured for each selected plant using a meter scale. After the tillers of the selected plants were counted and averaged to determine the TN, all green leaf blades were separated from the sampled plants, counted, and averaged to determine the GLN. Subsequently, all green leaf blades were run through an area meter (LI 3100; LI-COR Inc., Lincoln, NE, USA) to measure the green leaf area (GLA). The values of GLA per plant were divided by the ground area per plant to obtain the LAI. Finally, all parts of ten plants (leaves, stems, and spikes) were oven-dried at 80 °C until their weight became constant to determine the SDW. 

#### 4.3.2. Physiological Parameters

In parallel with the measurements of the morphological growth parameters, RWC and chlorophyll pigment contents (namely, Chl-a, Chl-b, and Chlt) were measured using the second leaf (from the top). Five leaves from each subplot were excised and an area of 15 cm^2^ from each leaf was excised, immediately weighed to record their fresh weight (FW), rehydrated in distilled water in the dark at 25 °C for 24 h to obtain their turgid weight (TW), and then dried at 80 °C until constant weight to record their dry weight (DW). Based on the values of FW, TW, and DW, the percentage of RWC was assessed using the following equation:

(1) $$\mathrm{RWC}\;\left(\%\right)=\frac{\mathrm{FW}-\mathrm{DW}}{\mathrm{TW}-\mathrm{DW}}\times 100$$

The methods of Arnon [62] and Lichtenthaler and Wellburn [63] were followed to determine the chlorophyll pigment contents. Fragments of fresh leaves (0.5 g) were collected from each subplot and soaked in 10 mL acetone (80%) and kept in the dark for a few days to extract the sap. After that, the extracted sap was centrifuged at 400 rpm for 10 min and the absorbance was read at 663 nm (A663) and 645 nm (A645) using a spectrophotometer (UV-2550, Shimadzu, Tokyo, Japan). The values of the absorbance reading were applied in the following formulas to calculate the concentrations of the different chlorophyll pigments in mg g^−1^ fresh weight (FW):

Chl a mg g^−1^ FW = [(12.7 × A663) − (2.69 × A645)] × V/(1000 × FW) (2)

Chl b mg g^−1^ FW= [(22.9 × A645) − (4.68 × A663)] × V/(1000 × FW) (3)

Chl t mg g^−1^ FW= [(20.2 × A645) + (8.02 × A663)] × V/(1000 × FW) (4)

where V is the volume of the extracted liquid.

#### 4.3.3. Yield Parameters and Water Productivity

At the maturity stage, which was recorded in the middle of April in both growing seasons, fifty spikes were randomly selected from each subplot to determine the different yield components (SL, GNS, GWS, and TGW). Thereafter, 2.1 m^2^ from each subplot (three inner rows of 3.5 m) were harvested and weighed to determine the BY. Subsequently, the plants were threshed and the grains were cleaned and weighed to determine the GY. After the values of BY and GY were converted to ton ha^−1^, the HI and WP were calculated by dividing the GY by BY and growing season irrigation water, respectively. 

### 4.4. Statistical Analysis

The normality distribution and variance homogeneity of the data for all parameters were assessed using Shapiro–Wilk and Bartlett’s chi-squared tests, respectively, before the analysis of variance (ANOVA) for both seasons. Since a uniform error variance was observed for the tested parameters in the two growing seasons, the combined analysis was performed on the data using the ANOVA appropriate for the split plot in a randomized complete block design across two growing seasons. Season and replication factors were considered random effects, while IR and Co-A treatments were considered fixed effects. The analysis was conducted using the CoStat computer software package for Windows (version 6.45, CoHort Software, VSN International Ltd., Oxford, UK). The significance of differences between the different treatments of each factor was performed based on the F-test. The differences among the mean values of IR, Co-A, and their interaction were separated according to post hoc test (Tukey’s test) at a 0.05 level of probability. A Pearson’s correlation coefficient was performed using XLSTAT computer software program statistical package (vers. 2019.1, Excel add-ins soft SARL, New York, NY, USA) to define the degree of correlation between all parameters across seasons, replications, and Co-A treatments under each IR treatment. A heatmap was performed using R statistical software (ver. 4.2.2, R Foundation for Statistical Computing, Vienna, Austria) to integrate all parameters with different Co-A treatments under each IR treatment. All figures were created using the Sigma Plot software program (ver. 14.0; SPSS, Chicago, IL, USA). 

## 5. Conclusions

In conclusion, LMI induced a significant reduction in numerous morpho-physiological attributes, which ultimately led to a significant reduction in the yield and yield components of wheat crop in arid climatic conditions. Interestingly, the exogenous co-application of plant nutrients, particularly micronutrients, and SA through the soil and foliar spray methods effectively curbed the negative effects of the LMI treatment, mainly by enhancing various morphological traits, RWC, and photosynthetic pigments (Chl-a, Chl-b, and Chlt), which ultimately improved the production and WP of wheat. This combination of plant nutrients and SA also played a vital role in enhancing the growth and production of wheat under NI conditions. In addition, the application of plant nutrients and SA through both soil and foliar spray was more effective than the application through foliar spray only under either NI or LMI conditions. Therefore, the heatmap cluster analysis identified S_SA_+ F_SA + Mic_ as the best treatment for enhancing all the studied parameters under both IR treatments, followed by S_SA + Mic_ + F_SA_ and F_SA + Mic_. Overall, given the lack of freshwater and poor soil fertility in arid and semiarid climates, the combined use of SA and plant nutrients, especially micronutrients, through the foliar and soil methods may be a feasible and user-friendly strategy for sustainable wheat production, obtaining high net returns, and dealing with deficit water stress in these climates.

## Figures and Tables

**Table 1:** Analysis of variance (F-test) and mean values of the different growth parameters of wheat regarding the effects of season (S), irrigation treatment (IR), co-application (Co-A) of salicylic acid (SA), macronutrients (Mac), and micronutrients (Mic) through foliar (F) and soil (S) application methods, and their interaction.

Source of Variation		PH	TN	GLN	LAI	SFW	SDW
**Season (S)**							
First season		77.11 ^a^	4.17 ^a^	4.89 ^a^	1.76 ^a^	15.05 ^a^	7.39 ^a^
Second season		74.04 ^a^	4.11 ^a^	4.34 ^a^	1.39 ^b^	14.77 ^a^	6.81 ^a^
Irrigation treatment (IR)							
Normal irrigation		80.16 ^a^	4.85 ^a^	5.86 ^a^	2.07 ^a^	17.70 ^a^	8.32 ^a^
Limited irrigation		70.99 ^b^	3.46 ^b^	3.37 ^b^	1.08 ^b^	12.12 ^b^	5.89 ^b^
Co-application treatments (Co-A)							
Control		73.69 ^c^	3.78 ^c^	3.86 ^d^	1.35 ^e^	13.36 ^d^	6.27 ^d^
F_SA + Mic_		75.97 ^ab^	4.25 ^ab^	4.90 ^ab^	1.65 ^bc^	15.69 ^ab^	7.44 ^b^
F_SA + Mac_		76.56 ^a^	4.08 ^b^	4.86 ^b^	1.61 ^c^	15.37 ^b^	7.21 ^b^
S_SA_+ F_Mic_		75.94 ^ab^	4.20 ^ab^	4.31 ^c^	1.47 ^d^	14.17 ^c^	6.82 ^c^
S_SA_+ F_SA + Mic_		77.03 ^a^	4.39 ^a^	5.06 ^a^	1.74 ^a^	15.95 ^a^	7.80 ^a^
S_SA + Mic_ + F_SA_		76.03 ^a^	4.22 ^ab^	4.87 ^b^	1.68 ^ab^	15.79 ^ab^	7.35 ^b^
S_SA + Mic_ + F_Mac + Mic_		73.81 ^bc^	4.17 ^ab^	4.44 ^c^	1.52 ^d^	14.04 ^c^	6.82 ^c^
ANOVA	df						
S	1	0.059 ^ns^	0.663 ^ns^	0.143 ^ns^	0.001 **	0.286 ^ns^	0.082 ^ns^
IR	1	<0.001 ***	<0.001 ***	<0.001 ***	<0.001 ***	<0.001 ***	<0.001 ***
IR × S	1	0.103 ^ns^	0.581 ^ns^	0.004 **	<0.001 ***	0.256 ^ns^	0.330 ^ns^
Co-A	6	0.015 *	0.009 **	<0.001 ***	<0.001 ***	<0.001 ***	<0.001 ***
Co-A × S	6	0.120 ^ns^	0.677 ^ns^	0.969 ^ns^	<0.001 ***	0.585 ^ns^	0.261 ^ns^
Co-A × IR	6	0.367 ^ns^	0.626 ^ns^	0.003 **	0.008 **	0.002 **	0.002 **
Co-A × IR × S	6	0.779 ^ns^	0.782 ^ns^	0.042 *	0.098 ^ns^	0.071 ^ns^	0.225 ^ns^

Means sharing the same letter for S, IR, and Co-A in a same column do not differ significantly at the 0.05 level according Tukey’s test. ns, *, **, and *** indicate non-significant and significant at *p* ≤ 0.05, 0.01, and 0.001 respectively, in F-tests. Abbreviations of PH, TN, GLN, LAI, SFW, and SDW are plant height (cm), tiller number per plant, green leaf number per plant, leaf area index, shoot fresh weight (g plant^−1^), and shoot dry weight (g plant^−1^), respectively.

**Table 2:** Analysis of variance (F-test) and mean values of the different physiological parameters of wheat regarding the effects of season (S), irrigation treatment (IR), the co-application (Co-A) of salicylic acid (SA), macronutrients (Mac), and micronutrients (Mic) through foliar (F) and soil (S) application methods, and their interaction.

Source of Variation		RWC	Chl-a	Chl-b	Chlt
**Season (S)**					
First season		69.57 ^b^	1.58 ^b^	0.76 ^b^	2.37 ^b^
Second season		75.05 ^a^	1.76 ^a^	0.97 ^a^	2.76 ^a^
Irrigation treatment (IR)					
Normal irrigation		81.15 ^a^	2.07 ^a^	1.08 ^a^	3.19 ^a^
Limited irrigation		63.48 ^b^	1.27 ^b^	0.65 ^b^	1.94 ^b^
Co-application treatments (Co-A)					
Control		67.57 ^d^	1.35 ^d^	0.71 ^d^	2.09 ^d^
F_SA + Mic_		75.03 ^a^	1.78 ^ab^	0.90 ^b^	2.71 ^b^
F_SA + Mac_		72.96 ^b^	1.77 ^b^	0.94 ^ab^	2.74 ^ab^
S_SA_+ F_Mic_		70.08 ^c^	1.58 ^c^	0.80 ^c^	2.41 ^c^
S_SA_+ F_SA + Mic_		75.51 ^a^	1.84 ^a^	0.97 ^a^	2.82 ^a^
S_SA + Mic_ + F_SA_		74.10 ^ab^	1.78 ^ab^	0.92 ^b^	2.73 ^b^
S_SA + Mic_ + F_Mac + Mic_		70.96 ^c^	1.62 ^c^	0.82 ^c^	2.45 ^c^
ANOVA	df				
S	1	0.009 **	0.004 **	0.008 **	0.005 **
IR	1	<0.001 ***	<0.001 ***	<0.001 ***	<0.001 ***
IR × S	1	0.002 **	<0.001 ***	<0.001 ***	<0.001 ***
Co-A	6	<0.001 ***	<0.001 ***	<0.001 ***	<0.001 ***
Co-A × S	6	0.260 ^ns^	<0.001 ***	<0.001 ***	<0.001 ***
Co-A × IR	6	<0.001 ***	<0.001 ***	<0.001 ***	<0.001 ***
Co-A × IR × S	6	0.758 ^ns^	0.321 ^ns^	0.022 *	0.248 ^ns^

Means sharing the same letter for S, IR, and Co-A in a same column do not differ significantly at the 0.05 level according to Tukey’s test. ns, *, **, and *** indicate non-significant and significant at *p* ≤ 0.05, 0.01, and 0.001 respectively, in F-tests. Abbreviations of RWC, Chl-a, Chl-b, and Chlt indicate relative water content (%), chlorophyll-a (mg g^−1^ FW), chlorophyll-b (mg g^−1^ FW), and total chlorophyll content (mg g^−1^ FW), respectively.

**Table 3:** Analysis of variance (F-test) and mean values of the different yield components, yield, and water productivity of wheat regarding the effects of season (S), irrigation treatment (IR), co-application (Co-A) of salicylic acid (SA), macronutrients (Mac), and micronutrients (Mic) through foliar (F) and soil (S) application methods, and their interaction.

Source of Variation		SL	GWS	GNS	TGW	GY	BY	HI	WP
Season (s)									
First season		8.27 ^a^	1.51 ^a^	42.74 ^a^	34.76 ^a^	5.18 ^a^	14.69 ^a^	34.60 ^a^	10.94 ^a^
Second season		8.35 ^a^	1.57 ^a^	43.65 ^a^	35.55 ^a^	5.10 ^a^	14.82 ^a^	33.83 ^a^	10.70 ^a^
Irrigation treatment (IR)									
Normal irrigation		9.05 ^a^	1.91 ^a^	49.03 ^a^	39.00 ^a^	6.52 ^a^	17.42 ^a^	37.43 ^a^	10.05 ^b^
Limited irrigation		7.57 ^b^	1.17 ^b^	37.35 ^b^	31.30 ^b^	3.76 ^b^	12.09 ^b^	31.00 ^b^	11.59 ^a^
Co-application treatments (Co-A)									
Control		8.22 ^ns^	1.35 ^d^	40.65 ^e^	32.25 ^d^	4.44 ^d^	13.46 ^d^	31.99 ^d^	9.05 ^e^
F_SA + Mic_		8.30 ^ns^	1.60 ^b^	43.66 ^bc^	36.18 ^a^	5.40 ^b^	15.41 ^ab^	34.52 ^abc^	11.43 ^b^
F_SA + Mac_		8.32 ^ns^	1.56 ^b^	43.83 ^bc^	35.05 ^b^	5.26 ^b^	14.99 ^b^	34.43 ^bc^	10.97 ^c^
S_SA_+ F_Mic_		8.18 ^ns^	1.47 ^c^	41.60 ^de^	34.74 ^bc^	4.93 ^c^	14.23 ^c^	33.97 ^c^	10.31 ^d^
S_SA_+ F_SA + Mic_		8.46 ^ns^	1.70 ^a^	45.55 ^a^	36.91 ^a^	5.66 ^a^	15.83 ^a^	35.31 ^a^	12.06 ^a^
S_SA + Mic_ + F_SA_		8.30 ^ns^	1.66 ^a^	44.58 ^ab^	36.92 ^a^	5.36 ^b^	15.10 ^b^	35.15 ^ab^	11.55 ^b^
S_SA + Mic_ + F_Mac + Mic_		8.38 ^ns^	1.47 ^c^	42.47 ^cd^	34.02 ^c^	4.94 ^c^	14.28 ^c^	34.14 ^c^	10.38 ^d^
ANOVA	df								
S	1	0.107 ^ns^	0.101 ^ns^	0.058 ^ns^	0.063 ^ns^	0.430 ^ns^	0.479 ^ns^	0.056 ^ns^	0.272 ^ns^
IR	1	<0.001 ***	<0.001 ***	<0.001 ***	<0.001 ***	<0.001 ***	<0.001 ***	<0.001 ***	<0.001 ***
IR × S	1	0.112 ^ns^	0.119 ^ns^	0.732 ^ns^	<0.001 ***	0.288 ^ns^	0.115 ^ns^	0.072 ^ns^	0.127 ^ns^
Co-A	6	0.637 ^ns^	<0.001 ***	<0.001 ***	<0.001 ***	<0.001 ***	<0.001 ***	<0.001 ***	<0.001 ***
Co-A × S	6	0.741 ^ns^	0.601 ^ns^	0.507 ^ns^	0.100 ^ns^	0.170 ^ns^	0.245 ^ns^	0.136 ^ns^	0.135 ^ns^
Co-A × IR	6	0.715 ^ns^	<0.001 ***	0.005 **	<0.001 ***	<0.001 ***	0.001 **	<0.001 ***	<0.001 ***
Co-A × IR × S	6	0.813 ^ns^	0.058 ^ns^	0.899 ^ns^	0.001^**^	0.740 ^ns^	0.105 ^ns^	0.058 ^ns^	0.542 ^ns^

Means sharing the same letter for S, IR, and Co-A in a same column do not differ significantly at the 0.05 level according to Tukey’s test. ns, **, and *** indicate non-significant and significant at *p* ≤ 0.01, and 0.001 respectively, in F-tests. Abbreviations of SL, GWS, GNS, TGW, GY, BY, HI, and WP are spike length (cm), grain weight per spike (g), grain number per spike, thousand-grain weight (g), grain yield (ton ha^−1^), biological yield (ton ha^−1^), harvest index (%), and water productivity (kg mm^−1^ ha^−1^), respectively.

**Table 4:** Pearson’s correlation coefficients between all the studied parameters of wheat under normal irrigation (upper right) and limited irrigation (lower left) treatments over both growing seasons (n = 42).

Parameters	1	2	3	4	5	6	7	8	9	10	11	12	13	14	15	16	17	18
PH (1)		0.34 ^ns^	0.32 ^ns^	0.28 ^ns^	0.39 ^ns^	0.36 ^ns^	0.45 ^ns^	0.23 ^ns^	0.42 ^ns^	0.33 ^ns^	−0.48 ^ns^	0.48 ^ns^	0.29 ^ns^	0.60 ^ns^	0.45 ^ns^	0.27 ^ns^	0.53 ^ns^	0.45 ^ns^
TN (2)	0.65 ^ns^		0.47 ^ns^	0.55 ^ns^	0.56 ^ns^	0.58 ^ns^	0.59 ^ns^	0.52 ^ns^	0.49 ^ns^	0.45 ^ns^	0.39 ^ns^	0.49 ^ns^	0.51 ^ns^	0.55 ^ns^	0.42 ^ns^	0.50 ^ns^	0.52 ^ns^	0.42 ^ns^
GLN (3)	0.84 *	0.65 ^ns^		0.97 ***	0.93 **	0.94 **	0.86 *	0.98 ***	0.88 **	0.98 ***	0.21 ^ns^	0.92 **	0.82 *	0.85 *	0.95 **	0.91 **	0.64 ^ns^	0.95 **
LAI (4)	0.78 *	0.68 ^ns^	0.97 ***		0.93 **	0.96 ***	0.92 **	0.98 ***	0.81 *	0.96 ***	0.26 ^ns^	0.93 **	0.81 *	0.88 **	0.93 **	0.91 **	0.59 ^ns^	0.93 **
SFW (5)	0.79 *	0.80 *	0.97 ***	0.95 **		0.90 **	0.90 **	0.91 **	0.94 **	0.96 ***	0.21 ^ns^	0.98 ***	0.91 **	0.86 *	0.94 **	0.89 **	0.56 ^ns^	0.94 **
SDW (6)	0.84 *	0.73 ^ns^	0.99 ***	0.98 ***	0.97 ***		0.95 **	0.92 **	0.81 *	0.91 **	0.22 ^ns^	0.91 **	0.84 *	0.81 *	0.98 ***	0.98 ***	0.66 ^ns^	0.98 ***
RWC (7)	0.86 *	0.76 *	0.99 ***	0.94 **	0.96 ***	0.97 ***		0.84 *	0.73 ^ns^	0.84 *	0.04 ^ns^	0.91 **	0.76 *	0.89 **	0.92 **	0.89 **	0.60 ^ns^	0.92 **
Ch a (8)	0.91 **	0.87 *	0.95 **	0.90 **	0.95 **	0.94 **	0.96 ***		0.85 *	0.98 ***	0.33 ^ns^	0.91 **	0.82 *	0.84 *	0.91 **	0.88 **	0.69 ^ns^	0.91 **
Ch b (9)	0.92 **	0.79 *	0.97 ***	0.93 **	0.94 **	0.96 ***	0.98 ***	0.99 ***		0.94 *	0.29 ^ns^	0.92 **	0.93 **	0.72 ^ns^	0.89 **	0.83 *	0.66 ^ns^	0.89 **
Cht (10)	0.92 **	0.84 *	0.96 ***	0.91 **	0.95 **	0.95 **	0.97 ***	1.00 ***	0.99 ***		0.30 ^ns^	0.96 ***	0.89 **	0.84 *	0.94 **	0.89 **	0.65 ^ns^	0.94 **
SL (11)	0.81 *	0.77 *	0.88 **	0.76 *	0.82 *	0.85 *	0.89 **	0.89 **	0.88 **	0.89 **		0.26 ns	0.51 ns	−0.08 ^ns^	0.19 ^ns^	0.31 ^ns^	−0.21 ^ns^	0.19 ^ns^
GWS (12)	0.79 *	0.77 *	0.98 ***	0.99 ***	0.98 ***	0.98 ***	0.96 ***	0.94 **	0.95 **	0.95 **	0.80*		0.92 **	0.89 **	0.94 **	0.88 **	0.60 ^ns^	0.94 **
NGPS (13)	0.79 *	0.72 ^ns^	0.98 ***	0.99 ***	0.96 ***	0.97 ***	0.97 ***	0.93 **	0.96 ***	0.94 **	0.79 *	0.99 ***		0.64 ^ns^	0.89 **	0.89 **	0.59 ^ns^	0.89 **
TGW (14)	0.79 *	0.83 *	0.97 ***	0.97 ***	0.98 ***	0.98 ***	0.94 **	0.95 **	0.95 **	0.95 **	0.81 *	0.99 ***	0.96 ***		0.81 *	0.69 ^ns^	0.59 ^ns^	0.81 *
GY (15)	0.81 *	0.81 *	0.99 ***	0.98 ***	0.97 ***	0.98 ***	0.98 ***	0.96 ***	0.97 ***	0.96 ***	0.85 *	0.99 ***	0.99 ***	0.99 ***		0.97 ***	0.64 ^ns^	1.00 ***
BY (16)	0.77 *	0.76 *	0.99 ***	0.97 ***	0.99 ***	0.98 ***	0.96 ***	0.93 **	0.93 **	0.93 **	0.84 *	0.98 ***	0.96 ***	0.98 ***	0.98 ***		0.56 ^ns^	0.97 ***
HI (17)	0.79 *	0.82 *	0.89 **	0.88 **	0.84 *	0.88 **	0.91 **	0.92 **	0.94 **	0.93 **	0.80 *	0.91 **	0.92 **	0.90* *	0.93 **	0.84 *		0.64 ^ns^
WP (18)	0.81 *	0.81 *	0.99 ***	0.98 ***	0.97 ***	0.98 ***	0.98 ***	0.96 ***	0.97 ***	0.96 ***	0.85 *	0.99 **	0.99 ***	0.99 ***	1.00 ***	0.98 ***	0.93 **	

Abbreviations of PH, TN, GLN, LAI, SFW, SDW, RWC, Chl-a, Chl-b, Chlt, SL, GWS, G NS, TGW, GY, BY, HI, and WP are plant height, tiller number per plant, green leaf number per plant, leaf area index, shoot fresh weight, shoot dry weight, relative water content, chlorophyll-a, chlorophyll-b, total chlorophyll content, spike length, grain weight per spike, grain number per spike, thousand-grain weight, grain yield per ha, biological yield per ha, harvest index, and water productivity, respectively. ns, non-significant, and *, **, and *** indicate significant at *p* ≤ 0.05, 0.01, and 0.001, respectively.

**Table 5:** Effects of the different co-applications (Co-A) of salicylic acid (SA), macronutrients (Mac), and micronutrients (Mic) through foliar (F) and soil (S) application methods on the cost of production and monetary efficiency of wheat under normal and limited irrigation conditions.

Co-Application Treatments (Co-A)	Cost of Cultivation (USD ha^−1^)				Increase Yield (ton ha^−1^)	Monetary Efficiency (USD ha^−1^)	
	Soil Fertilizer Cost	Foliar Fertilizer Cost	Foliar Machine Cost	Total Coats		Revenue (USD ha^−1^)	Profit (USD ha^−1^)
	**Normal Irrigation**						
Control	0.00	0.00	0.00	0.00	0.00	0.00	0.00
F_SA + Mic_	0.00	41.23	100	141.23	0.775	245.57	104.34
F_SA + Mac_	0.00	49.00	100	149.00	0.815	258.24	109.24
S_SA_ + F_Mic_	60.00	36.81	100	196.81	0.328	103.93	−92.88
S_SA_ + F_SA + Mic_	60.00	49.00	100	209.00	0.927	293.73	84.73
S_SA + Mic_ + F_SA_	165.3	4.24	100	269.54	0.459	145.44	−124.10
S_SA + Mic_ + F_Mac + Mic_	165.3	81.40	100	346.70	0.292	92.52	−254.18
	**Limited Irrigation**						
Control	0.00	0.00	0.00	0.00	0.00	0.00	0.00
F_SA + Mic_	0.00	41.23	100	141.23	1.165	369.14	227.91
F_SA + Mac_	0.00	49.00	100	149.00	0.837	265.21	116.21
S_SA_ + F_Mic_	60.00	36.81	100	196.81	0.652	206.59	9.78
S_SA_ + F_SA + Mic_	60.00	49.00	100	209.00	1.468	465.15	256.15
S_SA + Mic_ + F_SA_	165.3	4.24	100	269.54	1.387	439.48	169.94
S_SA + Mic_ + F_Mac + Mic_	165.3	81.40	100	346.70	0.720	228.14	−118.56

**Table 6:** Physicochemical properties of the soil collected from the study site during 2020–2021 (season 1) and 2021–2022 (season 2).

Physicochemical Properties	Season 1	Season 2
pH (soil paste 1:5)	7.86	7.83
Electrical conductivity (dS m^−1^)	3.60	3.67
Organic matter (%)	0.48	0.46
CaCO_3_ (%)	29.01	29.42
Nitrogen (%)	0.12	0.09
Phosphorus (mg kg^−1^)	9.4	11.70
Potassium (mg kg^−1^)	186.9	167.1
Manganese (mg kg^−1^)	4.83	5.24
Zinc (mg kg^−1^)	1.01	1.13
Water-holding capacity (%)	18.69	18.42
Permanent wilting point (%)	7.28	7.14
Sand (%)	57.92	56.70
Silt (%)	28.40	29.26
Clay (%)	13.68	14.04
Soil texture	Sandy loam	Sandy loam

## Data Availability

All data are presented within the article.

## References

[1] Grote U., Fasse A., Nguyen T.T., Erenstein O. (2021). Food security and the dynamics of wheat and maize value chains in Africa and Asia. Front. Sustain. Food Syst..

[2] (2022). FAOSTAT, Food and Agriculture Organization of the United Nations Statistics Database, Rome. http://www.fao.org/faostat/en/#data/QC.

[3] Sallam A., Alqudah A.M., Dawood M.F., Baenziger P.S., Börner A. (2019). Drought stress tolerance in wheat and barley: Advances in physiology, breeding and genetics research. Int. J Mol. Sci..

[4] Mohammed N., El-Hendawy S., Alsamin B., Mubushar M., Dewir Y.H. (2023). Integrating application methods and concentrations of salicylic acid as an avenue to enhance growth, production, and water use efficiency of wheat under full and deficit irrigation in arid countries. Plants.

[5] Chai Q., Gan Y., Zhao C., Xu H.L., Waskom R.M., Niu Y., Siddique K.H.M. (2016). Regulated deficit irrigation for crop production under drought stress. A review. Agron. Sustain. Dev..

[6] Kulkarni M., Soolanayakanahally R., Ogawa S., Uga Y., Selvaraj M.G., Kagale S. (2017). Drought response in wheat: Key genes and regulatory mechanisms controlling root system architecture and transpiration efficiency. Front. Chem..

[7] Hussain H.A., Hussain S., Khaliq A., Ashraf U., Anjum S.A., Men S., Wang L. (2018). Chilling and drought stresses in crop plants: Implications, cross talk, and potential management opportunities. Front. Plant Sci..

[8] Muhammad F., Raza M.A.S., Iqbal R., Zulfiqar F., Aslam M.U., Yong J.W.H., Altaf M.A., Zulfiqar B., Amin J., Ibrahim M.A. (2022). Ameliorating Drought Effects in Wheat Using an Exclusive or Co-Applied Rhizobacteria and ZnO Nanoparticles. Biology.

[9] Karim M.R., Rahman M.A. (2015). Drought risk management for increased cereal production in Asian least developed countries. Weather Clim. Extrem..

[10] Noctor G., Reichheld J.P., Foyer C.H. (2018). ROS-related redox regulation and signaling in plants. Semin. Cell Dev. Biol..

[11] Bakht S., Safdar K., Khair K., Fatima A., Fayyaz A., Ali S., Munir H., Farid M. (2020). The response of major food crops to drought stress: Physiological and biochemical responses. Agronomic Crops.

[12] Peng Z., Wang L., Xie J., Li L., Coulter J.A., Zhang R., Luo Z., Kholova J., Choudhary S. (2019). Conservation tillage increases water use efficiency of spring wheat by optimizing water transfer in a semi-arid environment. Agronomy.

[13] Cheng D., Wang Z., Yang L., Zhang L., Zhang Q. (2021). Combined effects of mulching and crop density on soil evaporation, temperature, and water use efficiency of winter wheat. Exp. Agric..

[14] El-Hendawy S., Alsamin B., Mohammed N., Al-Suhaibani N., Refay Y., Alotaibi M., Tola E., Mattar M.A. (2022). Combining planting patterns with mulching bolsters the soil water content, growth, yield, and water use efficiency of spring wheat under limited water supply in arid regions. Agronomy.

[15] Dutta T., Neelapu N.R., Wani S.H., Challa S. (2018). Compatible solute engineering of crop plants for improved tolerance toward abiotic stresses. Biochemical, Physiological and Molecular Avenues for Combating Abiotic Stress Tolerance in Plants.

[16] Hasanuzzaman M., Matin M., Fardus J., Hasanuzzaman M., Hossain M., Parvin K. (2019). Foliar application of salicylic acid improves growth and yield attributes by upregulating the antioxidant defense system in Brassica campestris plants grown in lead-amended soils. Acta Agrobot..

[17] Tayyab N., Naz R., Yasmin H., Nosheen A., Keyani R., Sajjad M., Hassan M.N., Roberts T.H. (2020). Combined seed and foliar pre-treatments with exogenous methyl jasmonate and salicylic acid mitigate drought induced stress in maize. PLoS ONE.

[18] Hafez E.M., Kheir A., Badawy S.A., Rashwan E., Farig M., Osman H.S. (2020). Differences in physiological and biochemical attributes of wheat in response to single and combined salicylic acid and biochar subjected to limited water irrigation in saline sodic soil. Plants.

[19] Ghosh U.K., Islam M.N., Siddiqui M.N., Khan M.A.R. (2021). Understanding the roles of osmolytes for acclimatizing plants to changing environment: A review of potential mechanism. Plant Signal. Behav..

[20] Sedaghat M., Sarvestani Z.T., Emam Y., Bidgoli A.M., Sorooshzadeh A. (2020). Foliar-applied GR24 and salicylic acid enhanced wheat drought tolerance. Russ. J. Plant Physiol..

[21] El Sherbiny H.A., El-Hashash E.F., Abou El-Enin M.M., Nofal R.S., Abd El-Mageed T.A., Bleih E.M., El-Saadony M.T., El-Tarabily K.A., Shaaban A. (2022). Exogenously applied salicylic acid boosts morpho-physiological traits, yield, and water productivity of lowland rice under normal and deficit irrigation. Agronomy.

[22] Khan M.I., Poor P., Janda T. (2022). Salicylic Acid: A versatile signaling molecule in plants. J. Plant Growth Regul..

[23] Karim M.R., Zhang Y.Q., Zhao R.R., Chen X.P., Zhang F.S., Zou C.Q. (2012). Alleviation of drought stress in winter wheat by late foliar application of zinc, boron, and manganese. J. Plant Nutr. Soil Sci..

[24] Wang Y., Zhang X., Chen J., Chen A., Wang L., Guo X., Niu Y., Liu S., Mi G., Gao Q. (2019). Reducing basal nitrogen rate to improve maize seedling growth, water and nitrogen use efficiencies under drought stress by optimizing root morphology and distribution. Agric. Water Manag..

[25] Ashraf M.Y., Tariq S., Saleem M., Khan M.A., Hassan S.W.U., Sadef Y. (2020). Calcium and zinc mediated growth and physiobiochemical changes in mung bean grown under saline conditions. J. Plant Nutr..

[26] Hassan M.U., Aamer M., Umer Chattha M., Haiying T., Shahzad B., Barbanti L., Nawaz M., Rasheed A., Afzal A., Liu Y. (2020). The critical role of zinc in plants facing the drought stress. Agriculture.

[27] Kumari V.V., Banerjee P., Verma V.C., Sukumaran S., Chandran M.A.S., Gopinath K.A., Venkatesh G., Yadav S.K., Singh V.K., Awasthi N.K. (2022). Plant nutrition: An effective way to alleviate abiotic stress in agricultural crops. Int. J. Mol. Sci..

[28] Bagci S.A., Ekiz H., Yilmaz A., Cakmak I. (2007). Effects of zinc deficiency and drought on grain yield of field-grown wheat cultivars in Central Anatolia. J. Agron. Crop Sci..

[29] Fageria N., Filho M.B., Moreira A., Guimarães C. (2009). Foliar fertilization of crop plants. J. Plant Nutr..

[30] Subbaiah L.V., Prasad T.N.V.K.V., Krishna T.G., Sudhakar P., Reddy B.R., Pradeep T. (2016). Novel effects of nanoparticulate delivery of zinc on growth, productivity, and zinc biofortification in maize (*Zea mays* L.). J. Agric. Food Chem..

[31] Walsh O.S., Shafian S., Christiaens R.J. (2018). Nitrogen fertilizer management in dryland wheat cropping systems. Plants.

[32] Ferrari M., Dal Cortivo C., Panozzo A., Barion G., Visioli G., Giannelli G., Vamerali T. (2021). Comparing soil vs. foliar nitrogen supply of the whole fertilizer dose in common wheat. Agronomy.

[33] Castro S.A.Q.d., Kichey T., Persson D.P., Schjoerring J.K. (2022). Leaf Scorching following Foliar Fertilization of Wheat with Urea or Urea–Ammonium Nitrate Is Caused by Ammonium Toxicity. Agronomy.

[34] Dass A., Rajanna G.A., Babu S., Lal S.K., Choudhary A.K., Singh R., Rathore S.S., Kaur R., Dhar S., Singh T. (2022). Foliar application of macro-and micronutrients improves the productivity, economic returns, and resource-use efficiency of soybean in a semiarid climate. Sustainability.

[35] Li H., Testerink C., Zhang Y. (2021). How roots and shoots communicate through stressful times. Trends Plant Sci..

[36] Kang J., Peng Y., Xu W. (2022). Crop root responses to drought stress: Molecular mechanisms, nutrient regulations, and interactions with microorganisms in the rhizosphere. Int. J. Mol. Sci..

[37] Amanullah M.I., Nabi H., Khalid S., Ahmad M., Muhammad A., Ullah S., Ali I., Fahad S., Adnan M., Elshikh S. (2021). Integrated foliar nutrients application improve wheat (*Triticum Aestivum* L.) productivity under calcareous soils in drylands. Commun. Soil Sci. Plant Anal..

[38] Mandre B.K., Singh R., Dubey M., Waskle U., Birla V. (2020). Effect of foliar application of nutrients on growth and yield attributing characters of black gram. Int. J. Curr. Microbiol. Appl. Sci..

[39] Mahmoodi B., Moballeghi M., Eftekhari A., Neshaie-Mogadam M. (2020). Effects of foliar application of liquid fertilizer on agronomical and physiological traits of rice (*Oryza sativa* L.). Acta Agrobot..

[40] Lv X., Ding Y., Long M., Liang W., Gu X., Liu Y., Wen X. (2021). Effect of foliar application of various nitrogen forms on starch accumulation and grain filling of wheat (*Triticum aestivum* L.) under drought stress. Front. Plant Sci..

[41] Wang S., Sun N., Yang S., Tian X., Liu Q. (2021). The effectiveness of foliar applications of different zinc source and urea to increase grain zinc of wheat grown under reduced soil nitrogen supply. J. Plant Nutr..

[42] Deswal J., Pandurangam V. (2018). Morpho-physiological and biochemical studies on foliar application of zinc, iron and boron in maize (*Zea mays* L.). J. Pharmacogn. Phytochem..

[43] Farooq M., Ullah A., Rehman A., Nawaz A., Nadeem A., Wakeel A., Nadeem F., Siddique K.H. (2018). Application of zinc improves the productivity and biofortification of fine grain aromatic rice grown in dry seeded and puddled transplanted production systems. Field Crops Res..

[44] Hussain S., Rao M.J., Anjum M.A., Ejaz S., Zakir I., Ali M.A., Ahmad N., Ahmad S., Hasanuzzaman M., Hakim K., Nahar K., Alharby H.F. (2019). Oxidative stress and antioxidant defense in plants under drought conditions. Plant Abiotic Stress Tolerance: Agronomic, Molecular and Biotechnological Approaches.

[45] Sharma P., Jha A.B., Dubey R.S., Pessarakli M., Pessarakli M. (2021). Reactive oxygen species generation, hazards, and defense mechanisms in plants under environmental (abiotic and biotic) stress conditions. Handbook of Plant and Crop Physiology.

[46] Naikwade P.V., Desai N.M., Patil M., Pawar U.R. (2023). Plant responses to drought stress: Morphological, physiological, molecular approaches, and drought resistance. Plant Metabolites under Environmental Stress: Mechanisms, Responses, and Adaptation Strategies.

[47] Vries F.T., Brown C., Stevens C.J. (2016). Grassland species root response to drought: Consequences for soil carbon and nitrogen availability. Plant Soil..

[48] Saud S., Fahad S., Yajun C., Ihsan M.Z., Hammad H.M., Nasim W., Amanullah J., Arif M., Alharby H. (2017). Effects of Nitrogen Supply on Water Stress and Recovery Mechanisms in Kentucky Bluegrass Plants. Front. Plant Sci..

[49] Tariq A., Pan K., Olatunji O.A., Graciano C., Li Z., Sun F., Zhang L., Wu X., Chen W., Song D. (2018). Phosphorous fertilization alleviates drought effects on *Alnus cremastogyne* by regulating its antioxidant and osmotic potential. Sci. Rep..

[50] Bechtaoui N., Rabiu M.K., Raklami A., Oufdou K., Hafidi M., Jemo M. (2021). Phosphate-dependent regulation of growth and stresses management in plants. Front. Plant Sci..

[51] Aksu G., Altay H. (2020). The effects of potassium applications on drought stress in sugar beet. Sugar Technol..

[52] Akhtar N., Ilyas N., Arshad M., Meraj T.A., Hefft D.I., Jan B.L., Ahmad P. (2022). The impact of calcium, potassium, and boron application on the growth and yield characteristics of durum wheat under drought conditions. Agronomy.

[53] Mostofa M.G., Rahman M.M., Ghosh T.K., Kabir A.H., Abdelrahman M., Khan M.A.R., Mochida K., Tran L.S.P. (2022). Potassium in plant physiological adaptation to abiotic stresses. Plant Physiol. Biochem..

[54] Parveen A., Ashraf M.A., Hussain I., Perveen S., Rasheed R., Mahmood Q., Hussain S., Ditta A., Hashem A., Al-Arjani A.F. (2021). Promotion of growth and physiological characteristics in water-stressed *Triticum aestivum* in relation to foliar-application of salicylic acid. Water.

[55] Maruri-López I., Aviles-Baltazar N.Y., Buchala A., Serrano M. (2019). Intra and extracellular journey of the phytohormone salicylic acid. Front. Plant Sci..

[56] Kimbembe R.E.R., Li G., Fu G., Feng B., Fu W., Tao L., Chen T. (2020). Proteomic analysis of salicylic acid regulation of grain filling of two near-isogenic rice (*Oryza sativa* L.) under soil drying condition. Plant Physiol. Biochem..

[57] Liu X., Meng F.X., Zhang S.Q., Lou C.H. (2003). Ca^2+^ is involved in the signal transduction during stomatal movement induced by salicylic acid in Viciafaba. Physiol. Mol. Biol. Plants..

[58] Bagautdinova Z.Z., Omelyanchuk N., Tyapkin A.V., Kovrizhnykh V.V., Lavrekha V.V., Zemlyanskaya E.V. (2022). Salicylic acid in root growth and development. Int. J. Mol. Sci..

[59] Alotaibi M., El-Hendawy S., Mohammed N., Alsamin B., Refay Y. (2023). Appropriate application methods for salicylic acid and plant nutrients combinations to promote morpho-physiological traits, production, and water use efficiency of wheat under normal and deficit irrigation in an arid climate. Plants.

[60] Allen R.G., Pereira L.S., Raes D., Smith M. (1998). Crop Evapotranspiration. Guidelines for Computing Crop Water Requirements.

[61] Zadoks J.C., Chang T.T., Konzak C.F. (1974). A decimal code for the growth stages of cereals. Weed Res..

[62] Arnon D.I. (1949). Copper enzymes in isolated chloroplasts. Polyphenoloxidase in *Beta vulgaris*. Plant Physiol..

[63] Lichtenthaler H.K. (1987). Chlorophylls and carotenoids: Pigments of photosynthetic biomembranes. Methods Enzymol..

